# Neurosyphilis Presenting as Intermittent Explosive Disorder and Acute Psychosis

**DOI:** 10.7759/cureus.6337

**Published:** 2019-12-10

**Authors:** Harneel S Saini, Matthew Sayre, Ishveen Saini, Nehad Elsharkawy

**Affiliations:** 1 Neurology, Allegheny General Hospital, Pittsburgh, USA; 2 Internal Medicine, Lewis Katz School of Medicine at Temple University, Philadelphia, USA; 3 Internal Medicine, Lake Erie College of Osteopathic Medicine (LECOM), Erie, USA; 4 Pharmacy, Lake Erie College of Osteopathic Medicine (LECOM), Bradenton, USA

**Keywords:** syphilis

## Abstract

We present a case of a patient with tertiary syphilis, manifesting as acute psychosis, auditory hallucinations and intermittent explosive disorder with pending legal ramifications for physical violence. Our patient had been seen and treated by a psychologist with Aripiprazole for his erratic and aggressive behavior coupled with his new found psychosis over a one-year period with no avail. Prior accounts of interaction with the patient described him as “easy going”, “laid back”, and cooperative. Our patient had a complete return to baseline mentation and functionality post treatment with 4 Million Units every four hours of penicillin for two weeks. Neurosyphilis is a disease that greatly affects the mental functioning capacity of those infected. While treatment of syphilis has become greatly straightforward, those living in impoverished conditions and without a continual access to the health care system can progress through the stages of syphilis. It is of vital importance to keep syphilis on our differential for patients with rapidly progressing and broadly encompassing psychiatric disturbances especially in patients that have a lower socioeconomic status.

## Introduction

Syphilis is a sexually transmitted disease caused by the gram-negative bacteria, Treponema pallidum. In structure, it is seen as a spiral microbe that is capable of causing debilitating effects on those infected such as certain neurological disorders and even death. Syphilis is observed in three stages categorized by varying levels of intensity [1]. Primary syphilis is usually observed as a single painless sore known as a chancre. This sore heals on its own within a couple of weeks. Secondary syphilis is seen as a rash that starts on the trunk of the body that eventually spreads across the entire body. The patient may experience fever, sore throat and fatigue [2]. If the patient is not treated properly for syphilis, the infection may become latent in which no symptoms are seen, but may continue to worsen and progress into the tertiary stage. Those infected in the tertiary stage may have damage caused to the brain, nerves, liver, and other organs. This stage is characterized by the bacterium infecting vital organs and can present as difficulty controlling voluntary muscle movement, vision problems and even dementia [1].

Perhaps the most debilitating and life altering outcome of an untreated syphilis infection is neurosyphilis in which the brain and spinal cord are affected. There are five classifications of neurosyphilis: asymptomatic, meningeal, meningovascular, general paresis and tabes dorsalis [1,2]. Asymptomatic neurosyphilis is the most common type in which no signs of neurological disease are seen. In meningeal neurosyphilis, the patient may experience stiff neck, nausea, and headaches as well as hearing loss or loss of vision. On the more critical end of the spectrum, meningovascular neurosyphilis is diagnosed if the patient experiences at least one stroke. The fourth classification of neurosyphilis, general paresis, can manifest as dementia, changes in personality and mood, weakening muscles, fatigue and paresis. Tabes dorsalis is diagnosed when the infection affects the spinal cord subsequently manifesting as ataxia, sensory and other motor disturbances [1-3].

## Case presentation

A 31-year-old Caucasian male presented to the emergency department in Northeastern Ohio, complaining of confusion and seeking treatment for a recently confirmed diagnosis of neurosyphilis. The patient’s new girlfriend, a healthcare professional, had recently been diagnosed with syphilis and hepatitis C, subsequently prompting the patient to get tested. As part of his diagnostic workup, the patient underwent a lumbar puncture and CSF-VDRL test (cerebrospinal fluid-venereal disease research laboratory test) resulting in a rapid plasma reagin (RPR) titer of 1:64 and VDRL titer of 1:256 which means he tested positive for Syphilis. He was sent to a nearby hospital for further treatment. After receiving one dose of 4 Million Units of intravenous penicillin, the patient became acutely agitated demanding to smoke. He was denied and subsequently became furious, lashed out at the faculty and left the hospital against medical advice. Early next morning, he presented to our hospital accompanied by his girlfriend in a combative and confused state. Upon questioning, the patient reported he had been diagnosed with intermittent explosive disorder with psychosis four months prior to current admission and was prescribed Aripiprazole. He also described a new onset of hearing voices which he described had no meaning or direction just a montage of irrelevant voices. He was particularly distressed by himself because he described himself as “calm” and “nice” but now could no longer control his anger and violent outbursts. On presentation he had a diffuse upper back and left lateral forearm maculopapular and non-blanching rash. Other symptoms included headache, fever, night sweats, 50 lb weight loss over three months, agitation, psychosis, neuropathy, diffuse tender lymphadenopathy and flank pain. He had penile and oral lesions several months ago that had resolved by the time of admission. The patient had a past medical history significant for intravenous drug use (IVDU) and 20 pack-year smoking history. The patient denied nausea, vomiting, diarrhea and constipation. On physical exam, the patient was afebrile, had cranial nerves 2 to 12 grossly intact, pupils accommodated and reacted bilaterally, no nystagmus present, muscle strength 5/5, deep tendon reflexes 2/4 and sensation bilaterally intact upper and lower extremities, no ataxia, negative dysdiadokinesia and finger to nose bilaterally. Heart and lungs were normal - no murmur, point of maximal intensity (PMI) was nondisplaced and no wheezes, crackles or rhonchi were auscultated. The patient had a negative HIV screen but a positive Hep C panel. Although MRI might have provided additional information, the patient was not agreeable to the testing. The patient was discharged, threatening to leave against medical advice (AMA), on IV penicillin G 4 Million Units every four hours for two weeks administered by his girlfriend and he was taken off the Aripiprazole. The patient and his girlfriend reported resolution of aggressive behavior and auditory hallucinations with return to baseline mentation.

## Discussion

Syphilis is known as the “The Great Imitator” because of its ability to mimic the symptoms of many other diseases. As a result, neurosyphilis can be a challenging diagnosis to make due to the overlap of symptoms and similarity of clinical features to other psychiatric disorders. This deception of presentation makes it no surprise that it is oftentimes misdiagnosed. Schizophrenia, dementia, and bipolar disorders, are all diagnoses that have found their way onto the diagnostic differential in a patient with neurosyphilis. In a study published by Lin et al., of 169 neurosphyilis patients the study recruited, 52 of the patients presented with psychiatric manifestations. These included cognitive impairment, personality disorders, delirium, hostility, dysarthria, paranoia, fecal and urinary incontinence, hallucinations and mania [4]. In 1992, Roberts and Emsley described neurosyphilis presentations. The most common presenting symptoms in patients of neurosyphilis were personality change in 76% of patients, memory dysfunction (62%), hostility (52%), hallucination (48%), delusion (19%), and interestingly enough only 12% of the patients had the classic neurological symptoms of Marcus Gunn decreased vibratory, motor weakness and ataxia. Diagnosis of “confirmed” neurosyphilis then relies on presentation of any stage of syphilis and a reactive CSF-VDRL [5]. Although the patient’s CSF analysis and clinical picture are consistent with a diagnosis of neurosyphilis, a coexisting psychiatric disorder (previous diagnosis of intermittent explosive disorder with psychosis) or a drug-induced psychosis/withdrawal syndromes (history of IVDU) could not be ruled out. The patient was being treated with Abilify for his diagnosed psychiatric disorder and during the reported admission, the patient remained aggressive, and indicated that he was also hearing voices. Personality changes and auditory hallucinations are all documented associations of late neurosyphilis, and their presence indicates that the disease itself has been present for a long period of time [6-7].

There is no consensus on treatment regimens for patients with neurosyphilis and psychotic features in terms of which neurotropic agents to use and at what dose. Hung et al. found that treatment with penicillin alone resolved their neurosyphilis (NS) patient’s auditory hallucinations and religious delusions [8]. In a case report documenting a 40-year-old male positive for neurosyphilis, antipsychotic treatment was attempted but was not found to be a beneficial measure [9]. However, in another case report 400 mg of valproate twice a day was used to reduce agitation symptoms effectively [10]. Of note, in the latter case study, penicillin was not effective in completely clearing the patient of psychotic symptoms. This unfortunate case may be indicative that some of those who eventually reach the state of neurosyphilis may never fully recover from the psychotic symptoms without additional antipsychotic medications. There have also been many cases described of using both anti-psychotic medications in combination with antibiotics. Sanchez and Zisselman described a series of five case reports of neurosyphilis patients treated effectively with anti-psychotics in combination with antibiotics [11-14]. Interestingly enough, our patient found Haloperidol suppressed his irritation and psychosis adequately and repeatedly requested it to help with masking his symptoms. Eventually our patient was taken off his prescription of Aripiprazole and appeared to have resolved his psychotic with penicillin alone.

## Conclusions

Syphilis is a sexually transmitted disease that has continued to be a challenge for health care providers. The World Health Organization estimates the incidence of new syphilis cases to be 6.1 million per year worldwide. Although syphilis can occur in stages, its spread to the central nervous system (CNS) can occur early or late in the disease. We present this case as a reminder of how important it is to consider neurosyphilis symptomatology even in a patient with a previous psychiatric diagnosis. The effects of delaying appropriate treatment or noncompliance from the patient can result in devastating effects and may even lead to death.
